# Relationship of foot pain with the increased risk of falls in patients with Parkinson’s disease

**DOI:** 10.1002/jfa2.70023

**Published:** 2024-12-12

**Authors:** Ana María Jiménez‐Cebrián, Francisco Javier Ruiz‐Sánchez, Marta Elena Losa‐Iglesias, Ricardo Becerro‐de‐Bengoa‐Vallejo, Daniel López‐López, Alonso Montiel‐Luque, Carmen de Labra, Miguel Ángel Saavedra‐García, Emmanuel Navarro‐Flores

**Affiliations:** ^1^ Department Nursing and Podiatry Faculty of Health Sciences Instituto de Investigación Biomédica de Málaga (IBIMA) University of Málaga Málaga Spain; ^2^ Research, Health and Podiatry Group Department of Health Sciences, Faculty of Nursing and Podiatry Industrial Campus of Ferrol Universidade da Coruña Ferrol Spain; ^3^ Faculty of Health Sciences Universidad Rey Juan Carlos Alcorcón Spain; ^4^ School of Nursing, Physiotherapy and Podiatry Universidad Complutense de Madrid Madrid Spain; ^5^ Department of Nursing and Podiatry Faculty of Health Sciences University of Málaga Málaga Spain; ^6^ Primary Health Care Centre San Miguel (Torremolinos), Health District Costa del Sol Málaga Spain; ^7^ Research, Health and Podiatry Group Department of Physiotherapy, Medicine and Biomedical Sciences, Faculty of Nursing and Podiatry Industrial Campus of Ferrol Universidade da Coruña Ferrol Spain; ^8^ Departamento de Educación Física y Deportiva Grupo INCIDE Universidade da Coruña A Coruña Spain; ^9^ Department of Nursing Faculty of Nursing and Podiatry Frailty Research Organizaded Group (FROG) University of Valencia Valencia Spain

**Keywords:** downton fall risk index, downton scale, fall risk, foot pain, Parkinson disease

## Abstract

**Introduction:**

Falls are one of the most frequent difficulties in patients with Parkinson's disease. The objective of this study was to determine the relationship between foot pain and the risk of falls in participants with Parkinson’s disease compared to a group of participants without Parkinson’s disease.

**Materials and Methods:**

The subjects (124) were divided into two groups, cases (*n* = 62) and controls (*n* = 62). They completed the Downton scale that collects the following 5 dimensions: previous falls, medications, sensory deficit, mental state, and ambulation.

**Results:**

Analyzing the Downton scale using dimensions, a significant difference was observed between both groups in all dimensions except mental state. Regarding the global result of risk of falls, the participants who had a diagnosis of Parkinson’s disease presented a high risk of falls, 40.3% compared to 3.2% of the non‐Parkinson’s disease group, with statistically significant differences (*p* < 0.05). For the numerical value of the Downton scale, there was a clear statistically significant difference between groups (2.65 ± 0.96 vs. 1.31 ± 1.19).

**Conclusion:**

This research confirms further evidence that people with Parkinson’s disease who suffer from foot pain are at high risk of falling, regardless of gender.

## 1 INTRODUCTION

Falls are one of the most frequent, recurrent, and relevant difficulties in people with Parkinson’s disease as the disease progresses [1]. Although the cause may be multifactorial, falls are attributed to the presence of motor symptoms such as bradykinesia, rigidity, stooped forward posture, and frozen gait. This vulnerability exposes them to other more serious injuries, and over time, increases their weakness and loss of independence [2, 3].

People with Parkinson’s disease have almost double the risk of falls and associated fractures than the general population of the same age [4]. The prevalence of Parkinson's disease in Spain shows geographical variations, with estimates ranging from 1280/100,000 to 270.24/100,000, and an estimated average of 682.2/100,000 [5]. This prevalence is similar to that observed in other European countries [6]. Globally, particularly in industrialized countries, prevalence increases with age: approximately 0.3% in the general population, 1.0% in individuals aged 60 years or older, and 3.0% in those over 80 years, in line with population aging [7].

In the study by Zejin Ou et al. (2021), the authors compared the prevalence rates of Parkinson’s disease across different countries from 1990 to 2019. They concluded that all countries showed an upward trend in Parkinson’s disease prevalence, attributed to population aging, with East Asia experiencing the highest increase, and Norway leading in Europe. The only region where Parkinson’s disease prevalence tended to decrease was Oceania [8].

Given the increasing prevalence of Parkinson’s disease globally, it is important to consider the associated risks, particularly the high incidence of falls among patients with Parkinson's disease. It is estimated that 76% of falls in patients with Parkinson's disease require medical attention and 33% result in fractures, with a mortality rate of 10.6% in these cases [9, 10, 11].

As cases of Parkinson’s disease continue to rise in developed countries, a corresponding increase in falls associated with this disease, posing a significant burden on health systems, is anticipated in the coming years [12]. Consequently, heightened awareness of this issue is imperative, necessitating the assessment of fall risk to formulate preventive strategies [13]. Moreover, controlling the risk factors linked to these falls is crucial, ultimately enhancing the quality of life for individuals affected by Parkinson’s disease [14].

The authors of a recent systematic review study [15] analyzed the predisposing factors of falls in patients with Parkinson’s disease, concluding that the following are prognostic factors with solid evidence: postural imbalance, walking alterations, and previous falls.

On the other hand, some studies indicate that the history of previous falls, along with the severity and duration of the disease, as well as the presence of dementia, are significant predictors [13]. All these circumstances are intrinsic and not modifiable. On the other hand, an influencing factor also to be considered, potentially modifiable, is the present foot pain and a very common symptom in patients with Parkinson’s disease [16, 17].

Research indicates that approximately 76% of patients with Parkinson’s disease experience pain, which can be classified into several types: musculoskeletal (34%–47%), dystonic (11%–23%), central neuropathic (19%–25%), radicular (19%–34%), and other types, including non‐radicular low back pain and arthritic pain [18]. Dystonic pain frequently affects the feet and is a common symptom in these patients, characterized by involuntary and sustained muscle contractions that lead to both pain and abnormal movements or postures of the foot. Additionally, foot dystonia can impair walking ability and balance further increasing the risk of falls [7, 19].

Previous studies have analyzed how kinesiophobia, or fear of movement, affects people with Parkinson’s disease [20], as well as the influences that the fear of falling may have on them [2]. Similarly, research has explored the correlation between leg muscle strength and the development of Parkinsonian gait [21], the association of hyponatremia with the presence of falls in these patients [22], and the risk of falls and its relationship with the presence of dysphagia [23]. Parkinson’s disease negatively impacts the foot and quality of life, mainly affecting overall foot health, reducing physical activity, social skills, and vigor [24]. The challenges of walking or moving, foot pain, and difficulties in foot hygiene and nail care as well as the concern about the deterioration in the condition of their feet, affect and decrease the quality of life of people with Parkinson’s disease. Regarding their self‐perception of their foot condition, patients with Parkinson’s disease perceive a poorer foot health compared to patients without Parkinson’s disease. Podiatric issues in Parkinson’s disease have a significant impact on reducing foot health‐related quality of life [25]. However, the relationship between fall risk and foot pain in Parkinson’s disease has not been investigated.

Therefore, we hypothesize that the presence of foot pain in patients with Parkinson’s disease influences an increase in the risk of falls. Thus, the objective of this study was to determine the relationship between foot pain and the risk of falls in patients with Parkinson’s disease compared to a group of patients without Parkinson’s disease.

## 2 METHODS

### 2.1 Design and sample

An observational descriptive and case‐control study was carried out from October to December 2020 in an institution for people with Parkinson’s disease in Malaga (Spain). The participants in the control group were recruited from the same locality as the cases. The study was completed by 124 subjects divided into two groups, a case group or participants with Parkinson’s disease (*n* = 62) and a control group or those without a Parkinson’s disease diagnosis (*n* = 62). Control group participants reported their medical conditions and medications to ensure they were free of any medical diagnoses that could potentially influence their risk of falls.

They were included by consecutive sampling using a simple successive procedure and without randomization. The informed consent was signed by all the members.

The additional inclusion criteria for both groups were as follows: adults between 50 and 84 years of age (age group where this disease is more prevalent) [26] and who could walk even with technical aids according to the Hoehn and Yahr scale [27, 28].

The exclusion criteria for both groups were as follows: adults without intact cognition according to the Parkinson's Disease‐Cognitive Rating Scale (PD‐CRS), who did not understand the instructions of the study or unable to provide informed consent, history of foot and/or ankle surgery, presence of peripheral neuropathy and severe orthopedic problems in the lower limbs. Both the inclusion and exclusion criteria were the same for both the case and control groups. Cases and controls were matched for age, gender, and body mass index (BMI).

### 2.2 Procedure

Initially, the researcher interviewed the participant, recording the questions associated with demographic and physical characteristics as well as the state of the disease. These include: sex, age, weight, height, and BMI calculated by the Quetelet index as kg/m^2^ [29]. Additionally, variables related to the disease included the presence of foot pain (yes or no), intensity (graded from 0 to 5, ranging from None to Severe) and frequency of foot pain (graded from 0 to 5, ranging from Never to Always), years of Parkinson’s disease (less than 5 years, between 6 and 10 years, between 11 and 15 years, between 16 and 20 years, and more than 20 years), and Hoehn and Yahr stage of Parkinson’s disease (stages ranging from stage 1 to stage 5, indicating the extent of the disease's progression, from less affected to most affected) [30]. For height measurement, we used a digital stadiometer that provides precise and calibrated measurements; this was the “Seca 274i”. Weight measurement was conducted using an electronic scale that provides accurate measurements of body weight; for this purpose, we used the “Omron Body Composition Monitor”. Both the intensity and frequency of pain were assessed using a Likert‐type scale.

#### 2.2.1 Measurement instruments

##### The Parkinson's Disease‐Cognitive Rating Scale

Next, the participants were administered the Parkinson's PD‐CRS [31], which is a tool specifically designed and validated for assessing the intact cognition in patients with Parkinson’s disease, addressing both frontal–subcortical and cortical domains and indicated by the International Parkinson and Movement Disorders Society as a global cognitive assessment instrument for Parkinson’s disease [32]. It focuses on areas such as memory, attention, executive function, and language. The scores on this scale are divided into the following areas: Total PD‐CRS Score: This is the sum of the item scores assessing both frontal–subcortical and cortical functions. The score ranges are as follows. Cognitively intact: This group consists of patients without cognitive impairment (highest scores). Mild cognitive impairment: This group presents subtle cognitive deficits, mainly in frontal–subcortical areas and some cortical dysfunctions (intermediate scores). Parkinson’s disease dementia: This group exhibits more advanced cognitive decline, characterized by a combination of frontal–subcortical and cortical dysfunctions (lowest scores). The items on the scale show a strong correlation with neuropsychological tests that assess frontal–subcortical and cortical cognitive domains. The scale has demonstrated excellent reliability, with an intraclass correlation coefficient (ICC) > 0.70 for the total PD‐CRS scores. The PD‐CRS also exhibited high internal consistency (Cronbach's *α* = 0.80) [31].

##### The Downton fall risk scale

Subsequently, the Downton fall risk scale, translated into Spanish [33], and also known as the Downton fall risk index, was administered. Several studies have examined the external validity of the Spanish translation of this tool [34, 35]. On the other hand, the Downton scale demonstrates a Cronbach's alpha reliability of 0.747, a test–retest ICC of 0.653, and the sensitivity for the prediction of falls is 92.2% (47/51: *p* < 0.001) [36]. The Downton scale gathers in 5 dimensions the factors with the greatest impact on the risk of falls: previous falls, medicines, sensory deficit, mental state, and ambulation. The minimum score can be 0 and the maximum 14 [37]. When the global score is equal to 0, there is no risk of falls; if it is equal to 1, the risk of falls is very low; if the total score is equal to 2, the risk of falls is low; if it is 3, the risk of falls is high; if the overall result is 4, the risk of falls is very high; and if it is 5, the risk of falls is extreme or severe [33].

### 2.3 Ethical considerations

The Bioethics and Biosafety Committee of the University of Valencia (Spain, 2020) approved this study (approval number 1450610). The participants signed the written consent before joining the research. The ethical and human experimentation standards of the Declaration of Helsinki (World Medical Association) and other organizations were always respected.

### 2.4 Sample size calculation

For the sample size, the Epidat 4.2 Program (Consellería de Sanidade, Xunta de Galicia, Spain; Pan American Health Organization, Universidad CES, Colombia) was used, calculating with specific levels of confidence, power, and groups of equal size. The total sample size was set at 124 participants (62 in each group) with a confidence level of 70%, a power of 0.80, an odds ratio of 2.0, and an expected exposure proportion of 66.67% in the group of cases as well as 50% in the control group.

### 2.5 Statistical analysis

For statistical analysis, SPSS 25.0v software (IBM Corp., Armonk, NY, USA) was used with an alpha error of 0.05 for a 95% confidence interval. Kolmogorov–Smirnov test was used to assess normality in quantitative data. Between‐group comparisons were examined using both the Student's *t*‐test and the Mann–Whitney *U*‐test with independent samples. Frequencies and percentages were assigned for categorical data, and differences between groups were compared using the chi‐squared test. The bivariate correlation between foot pain and falls was studied using the Spearman correlation coefficient.

## 3 RESULTS

### 3.1 Descriptive data

The sample of participants who completed the study consisted of 124 participants, participants without Parkinson’s disease (control group, *n* = 62) and participants with Parkinson’s disease (case group, *n* = 62), with an age range between 50 and 84 years. For descriptive data, no statistically significant differences were found between both groups (*p* > 0.05). It is shown in Table 1.

**TABLE 1:** Demographic and descriptive data of the Parkinson’s disease group and non‐Parkinson’s disease group.

Demographic and descriptive data		Total group Mean ± SD (range) (*n* = 124)	Non‐Parkinson’s disease group Mean ± SD (range) (*n* = 62)	Parkinson’s disease group Mean ± SD (range) (*n* = 62)	*p*‐Value
Age (years)		69.18 ± 9.12 (50–84)	69.13 ± 9.15 (50–84)	69.23 ± 9.15 (50–84)	0.097†
Weight (kg)		74.10 ± 14.84 (43–135)	74.83 ± 11.49 (54–100)	73.36 ± 17.63 (43–135)	0.582†
Height (m)		1.67 ± 0.09 (1.47–1.91)	1.67 ± 7.80 (1.47–1.85)	1.66 ± 9.64 (1.47–1.91)	0.690†
BMI (kg/m^2^)		26.61 ± 4.61 (16.16–40.31)	26.85 ± 3.90 (19.83–35.43)	26.37 ± 5.24 (16.16–40.31)	0.0563†
Sex (%)	Male	75 (60.5%)	38 (61.3%)	38 (61.3%)	0.854‡
	Female	49 (39.5%)	24 (38.7%)	24 (38.7%)	
Foot pain presence		85 (68.5%)	26 (41.9%)	59 (95.2%)	**<0.001‡**
Foot pain intensity	None	39 (31.5%)	36 (58.1%)	3 (4.8%)	**<0.001‡**
	Very slight	32 (25.8%)	16 (25.8%)	16 (25.8%)	
	Slight	27 (21.8%)	10 (16.1%)	17 (27.4%)	
	Moderate	24 (19.4%)	0 (0.0%)	24 (38.7%)	
	Severe	2 (1.6%)	0 (0.0%)	2 (3.2%)	
Foot pain frequency	Never	39 (31.5%)	36 (58.1%)	3 (4.8%)	**<0.001‡**
	Occasionally	44 (35.5%)	18 (29.0%)	26 (41.9%)	
	Many times	26 (21.0%)	8 (12.9%)	18 (29.0%)	
	Very often	10 (8.1%)	0 (0.0%)	10 (16.1%)	
	Always	4 (12%)	0 (0.0%)	5 (8.1%)	

*Note*: In all the analyses *p* < 0.05 (with a 95% confidence interval) was considered statistically significant. Mean ± standard deviation, range (min–max) and † Student's *t*‐test for independent samples were applied. ‡ Chi‐squared test was used.

Abbreviations: BMI, body mass index; SD, standard deviation.

Regarding the years diagnosed with Parkinson’s disease, 20.6% had a disease diagnosis for less than 5 years, 27.8% between 6 and 10 years, 29.4% between 11 and 15 years, 20.4% had between 16 and 20 years, and 1.8% of the sample had been suffering from Parkinson's for more than 20 years. According to the Hoehn and Yahr Scale [30], 14.5% of patients with Parkinson's disease were in stage 1, 30.6% in stage 2, 28.45% in stage 3, 9.7% in stage 4, and none were at stage 5.

### 3.2 Outcome measurements

Analyzing the Downton scale using dimensions, a statistically significant difference (*p* < 0.05) was observed between both groups in all its dimensions except mental state (Table 2).

**TABLE 2:** Comparisons of results of Downton fall risk scale by dimensions between the Parkinson’s disease group and non‐Parkinson’s disease group.

Downton fall risk scale		Non‐Parkinson’s disease group *n* (%)	Parkinson’s disease group *n* (%)	*p*‐Value
Previous falls	No	33 (53.2%)	22 (35.5%)	0.047*
	Yes	29 (46.8%)	40 (64.5%)	
Medicines	None	28 (45.2%)	0 (0%)	<0.001*
	Tranquilizers or sedatives	27 (43.5%)	61 (98.4%)	
	Diuretics	7 (11.3%)	1 (1.6%)	
	Hypotensive (nondiuretic)	0 (0%)	0 (0%)	
	Antiparkinsonians	0 (0%)	0 (0%)	
	Antidepressant	0 (0%)	0 (0%)	
	Other medications	0 (0%)	0 (0%)	
Sensory deficits	None	50 (80.6%)	18 (29%)	<0.001*
	Visual alterations	12 (19.4%)	44 (71%)	
	Hearing alterations	0 (0%)	0 (0%)	
	Extremities (stroke, etc)	0 (0%)	0 (0%)	
Mental state	Oriented	58 (93.5%)	57 (91.9%)	0.729
	Confused	4 (6.5%)	5 (8.1%)	
Ambulation	Normal	61 (98.4%)	45 (72.69%)	<0.001*
	Safe with help	1 (1.6%)	17 (27.4%)	
	Insecure with help	0 (0%)	0 (0%)	
	Impossible	0 (0%)	0 (0%)	

*Note*: Downton scale. Frequency, percentage (%) and Chi‐squared test (χ^2^) were utilized. Downton scale domains were divided as follows: (1) previous falls, (2) medicines, (3) sensory deficits, (4) mental state, and (5) ambulation. In all the analyses, *p* < 0.05 (with a 95% confidence interval) was considered statistically significant (**bold**).

Regarding the global result of risk of falls, the participants with Parkinson’s disease presented worse results on the Downton scale, with a high risk of falls in 40.3% compared to 3.2% in the group without Parkinson’s disease, with statistically significant differences (*p* < 0.05) between both groups (Table 3).

**TABLE 3:** Comparisons of risk of falls between the Parkinson’s disease group and non‐Parkinson’s disease control group obtained with Downton scale.

Outcomes measurements		Non‐ Parkinson’s disease group *n* (%)	Parkinson's disease group *n* (%)	*p*‐Value
Risk of falls	None	20 (32.3%)	0 (0.0%)	<0.001*
	Very low	15 (24.2%)	8 (12.9%)	
	Low	20 (32.3%)	18 (29%)	
	High	2 (3.2%)	25 (40.3%)	
	Very high	5 (8.1%)	10 (16.1%)	
	Severe	0 (0.0%)	1 (1.6%)	

*Note*: In all the analyses, *p* < 0.05 (with a 95% confidence interval) was considered statistically significant (**bold**).

Similarly, the numerical value of the Downton scale showed a clear statistically significant difference between the two groups, with the mean value of Parkinson’s disease group being higher (indicating a higher risk) than that of non‐Parkinson’s disease group (2.65 ± 0.96 vs. 1.31 ± 1.19) as shown in Table 4.

**TABLE 4:** Downton values in total group and each group.

Outcomes measurements	Total group Mean ± SD (range) (*n* = 124)	Non‐Parkinson’s group Mean ± SD (range) (*n* = 62)	Parkinson’s disease group Mean ± SD (range) (*n* = 62)	*p*‐Value
Downton value	1.98 ± 1.27 (0–5)	1.31 ± 1.19 (0–4)	2.65 ± 0.96 (1–5)	**<0.001†**

*Note*: In all the analyses, *p* < 0.05 (with a 95% confidence interval) was considered statistically significant. Mean ± standard deviation, range (min–max) and † Student's *t*‐test for independent samples were applied.

Abbreviation: SD, standard deviation.

The values obtained for the risk of falls due to foot pain, intensity, and frequency of foot pain are presented in Table 5.

**TABLE 5:** Comparison of the values of the risk of falls between values of foot pain, foot pain intensity, and foot pain frequency in the Parkinson’s disease group and non‐Parkinson’s disease group.

Risk of falls	People with Parkinson's disease group *n* = 62						Non‐Parkinson’s disease group *n* = 62						*p*‐Value
	None *n* (%)	Very low *n* (%)	Low *n* (%)	High *n* (%)	Very high *n* (%)	Severe *n* (%)	None *n* (%)	Very low *n* (%)	Low *n* (%)	High *n* (%)	Very high *n* (%)	Severe *n* (%)	
Foot pain													
No	0 (0%)	1 (1.6%)	1 (1.6%)	0 (0%)	1 (1.6%)	0 (0%)	13 (21%)	10 (16.1%)	10 (16.1%)	1 (1.6%)	2 (3.2%)	0 (0%)	**<0.001**
Yes	0 (0%)	7 (11.3%)	17 (27.4%)	25 (40.3%)	10 (16.1%)	0 (0%)	7 (11.3%)	5 (8.1%)	10 (16.1%)	1 (1.6%)	3 (4.8%)	0 (0%)
Foot pain intensity													
None	0 (0%)	1 (1.6%)	1 (1.6%)	0 (0.0%)	1 (1.6%)	0 (0%)	13 (21%)	10 (16.1%)	10 (16.1%)	1 (1.6%)	2 (3.2%)	0 (0%)	**<0.001**
Very slight	0 (0%)	1 (1.6%)	4 (6.5%)	6 (9.7%)	5 (8.1%)	0 (0%)	5 (8.1%)	3 (4.8%)	5 (8.1%)	0 (0%)	3 (4.8%)	0 (0%)
Slight	0 (0%)	2 (3.2%)	5 (8.1%)	7 (11.3%)	3 (4.8%)	0 (0%)	2 (3.2%)	2 (3.2%)	5 (8.1%)	1 (1.6%)	0 (0%)	0 (0%)
Moderate	0 (0%)	4 (6.5%)	6 (9.7%)	12 (19.4%)	2 (3.2%)	0 (0%)	0 (0%)	0 (0%)	0 (0%)	0 (0%)	0 (0%)	0 (0%)
Severe	0 (0%)	0 (0.0%)	2 (3.2%)	0 (0.0%)	0 (0.0%)	0 (0%)	0 (0%)	0 (0%)	0 (0%)	0 (0%)	0 (0%)	0 (0%)
Foot pain frequency													
Never	0 (0%)	1 (1.6%)	1 (1.6%)	0 (0.0%)	1 (1.6%)	0 (0%)	13 (21%)	10 (16.1%)	10 (16.1%)	1 (1.6%)	2 (3.2%)	0 (0%)	**<0.001**
Occasionally	0 (0%)	2 (3.2%)	9 (14.5%)	9 (14.5%)	6 (9.7%)	0 (0%)	5 (8.1%)	4 (6.5%)	6 (9.7%)	1 (1.6%)	2 (3.2%)	0 (0%)
Many times	0 (0%)	3 (4.8%)	4 (6.5%)	7 (11.3%)	4 (6.5%)	0 (0%)	2 (3.2%)	1 (1.6%)	4 (6.5%)	0 (0%)	1 (1.6%)	0 (0%)
Very often	0 (0%)	1 (1.6%)	1 (1.6%)	8 (12.90%)	0 (0.0%)	0 (0%)	0 (0%)	0 (0%)	0 (0%)	0 (0%)	0 (0%)	0 (0%)
Always	0 (0%)	1 (1.6%)	3 (4.8%)	1 (1.6%)	0 (0.0%)	0 (0%)	0 (0%)	0 (0%)	0 (0%)	0 (0%)	0 (0%)	0 (0%)

*Note*: Frequency. Percentage (%) and Chi‐squared test (χ2) were utilized. In all the analyses, *p* < 0.05 (with a 95% confidence interval) was considered statistically significant (bold).

Specifically, high or very high fall risk values were found in participants with Parkinson’s disease with foot pain in 56.4%, in 22.6% whose intensity of pain was moderate and when the frequency of pain was considered occasionally in 24.2%. After conducting a correlation study, we obtained a Spearman coefficient of 0.938 (*p* < 0.001), indicating a very strong positive relationship between foot pain and falls in participants with Parkinson’s disease. This suggests that, overall, as foot pain increases, the number of falls also significantly increases and vice versa (see Table 6).

**TABLE 6:** Comparison of Downton values between tranquilizers/sedatives medication in the Parkinson’s disease and non‐Parkinson’s disease group.

	Non‐Parkinson’s disease group	Parkinson's disease group
Downton value	Tranquilizers/sedatives	Tranquilizers/sedatives
0 (lower risk of falls)	0	8
1	8	17
2	16	25
3	1	10
4 (higher risk of falls)	2	1
	27 (43.5%)	61 (98.4%)

## 4 DISCUSSION

The objective of this study was to determine the risk of falls in participants with Parkinson’s disease using the Downton scale and its relationship with foot pain compared to a group of participants without Parkinson's disease. The findings corroborate the initial hypothesis, confirming a higher risk of falls in participants with Parkinson’s disease and highlighting that foot pain contributes to an increased risk of falls.

The results obtained with the Downton scale show that, in the group of participants with Parkinson’s disease, 40.3% have a high risk of falling, while in the group of participants without Parkinson’s disease it was 3.2% of them who have said high risk of falling falls. Several studies have investigated the risk of falls in people with Parkinson’s disease [28, 38, 39], but none of these have been like the present study, in which a case‐control study was conducted with participants with and without Parkinson’s disease, who were matched based on age, sex, and BMI.

In the study by Paker (2015), gait speed and the relationship with the risk of falls, measured with the Downton scale, were investigated. They obtained a negative correlation between walking speed and the Downton scale, which means that the lower the walking speed, the greater the risk of falls registered with this tool. In this study, the mean value (SD) obtained on the Downton scale was 3.86 (1.8), while in our study for patients with Parkinson's disease it was 2.65 (0.96), coinciding that, in both cases, there is a risk of falls in the group of patients with Parkinson's disease. The difference in fall rates between our study and that of Paker (2015) can be attributed to several factors. In our research, 98.4% of participants with Parkinson’s disease were taking tranquilizers or sedatives, which is known to increase the risk of falls [28]. In contrast, this factor is less prevalent in the Paker (2015) sample. Additionally, we included the variable of foot pain, a significant contributor to fall risk that was not previously considered.

However, Wilczynski (2021) [38], used the Tinetti Test to measure the risk of falls. The Tinetti Test, also known as the Performance Oriented Mobility Assessment, evaluates gait and balance, particularly in older adults. The assessment consists of gait assessment, (how a person initiates walking, their steadiness, speed control, and ability to turn) and balance assessment, (balance while sitting, standing, and during movement). Lower scores indicate a higher likelihood of falling. Using this test determined the risk of falls in patients with Parkinson’s disease and an association with the level of independence, also finding an inverse relationship between the level of independence and the risk of falls [40].

Lastly, the study by Sebastiá‐Amat et al. [39], used the Timed Up and Go test to measure the risk of falls, assessing the cognitive level and the risk of falls, with both being directly related. In turn, all also agree that the risk of falls is high in patients with Parkinson's disease. On the other hand, analyzing the dimensions of the Downton scale, as important risk factors for falls, it can be seen that in the group of participants with Parkinson’s disease, 64.5% experienced some “previous falls”. LeWitt (2020) and Grimbergen (2013) affirm that a history of previous falls increases the risk of subsequent falls, which in turn adversely affects the quality of life of individuals with Parkinson's disease, because of the fear of falling. Also, a previous study for patients with Parkinson's investigated kinesiophobia, or fear of moving [17], suggesting that kinesiophobia may be related to the fear of falling reported by LeWiit (2020) and Grimbergen (2013).

Likewise, it is important to note that 98.4% of the participants with Parkinson's disease in our study were taking some “sedative or tranquilizer” as medication. LeWitt (2020) exposes these drugs in a list of medications that favor and increase the risk of falls, primarily attributing it as a major factor in the increase in orthostatic hypotension and consequently, the risk of fainting and falls. In the planning of pharmacological treatment for these patients, LeWitt (2020) recommends a thorough review of the quantity and dose reduction of medications, especially those commonly prescribed.

In addition, our study revealed that the most prevalent sensory disturbance in the Parkinson’s disease group was “visual disturbances,” affecting 71% of participants compared to 19.4% in the non‐Parkinson’s disease group. In relation to this problem, Weil et al. [41] conducted a literature review on the visual disturbances present in Parkinson’s disease. They highlighted that people with Parkinson's disease experience changes in color vision, contrast sensitivity, and difficulties in complex visual tasks such as emotion recognition. They further noted that this visual dysfunction frequently coexists with postural instability and gait disorder, thus not only posing a risk of falls due to poor vision but also increasing the risk through its association with postural and gait disorders increases it.

However, it should be noted that, in our study, in the “mental state” dimension of the Downton scale there is no statistically significant difference between both groups, predominantly “oriented”, whose explanation is given to the fact that it was an exclusion criterion to suffer from cognitive disorders, which justifies this result.

With all this evidence, it can be deduced that the risk of falls in people with Parkinson’s disease is multifactorial. Therefore, it is crucial to reconsider a wide range of etiologies that can contribute to falls in these patients [4].

Our study examines foot pain frequency and intensity. Findings suggests that participants in the Parkinson’s disease group reported greater foot pain compared to the control group. The study also found that participants in the Parkinson’s disease group were at higher risk of falls when they also experienced foot pain (compared to participants with Parkinson’s disease who did not report foot pain). Additionally, we found that the intensity of foot pain was higher in the Parkinson’s disease group, and the risk of falls in these participants was elevated particularly when the pain intensity was moderate. Furthermore, we observed a high and very high risk of falls among participants with Parkinson’s disease at different levels of pain frequency.

Finally, our analysis revealed an association between foot pain and falls. It is important to highlight that, given the strength of this correlation, we further explored the relationship between these two variables using the Downton scale. Our findings revealed that the association between pain and falls was direct and not attributable to chance or random variation.

Like us, but with different approaches, previous studies in people with Parkinson’s disease considered the importance of foot health and hygiene, the proper use of footwear and functional mobility to slow down the progression of the disease [16, 24, 25, 42, 43, 44]. With this, it is corroborated that people with Parkinson’s disease experiencing foot problems, foot pain, intensity, and high frequency.

Similarly, but not specifically in Parkinson’s disease, Awale et al. [45] studied the relationship between foot pain and the risk of falls in a cohort of older adults and found that in the presence of foot pain the risk of falls doubled. With all this, we want to confirm that, in the risk of falls, it is important and crucial to assess foot health, and manage, as far as possible, foot pain.

Since Parkinson’s disease is different for each person, the pain in their feet and the changes in their feet are also different. The presence of a podiatrist is recommended in the multidisciplinary team that supports people with Parkinson’s disease to treat foot pathologies [46].

This study had some limitations. For future studies, it would be beneficial to combine measurements of foot pain with existing fall prediction models to yield more conclusive results. Also, simple random sampling would ensure a more uniform sample. Lastly, evaluating the results with a longitudinal follow‐up would determine more definitive and decisive conclusions.

## 5 CONCLUSIONS

This research provides evidence of foot pain and its relationship with the degree of fall risk and Parkinson’s disease. Participants with Parkinson’s disease exhibited a higher risk of falls than people without Parkinson’s disease, which was further influenced by the presence, intensity, and frequency of foot pain.

## AUTHOR CONTRIBUTIONS

**Ana María Jiménez‐Cebrián:** Conceptualization; methodology; formal analysis; data curation; writing—review and editing. **Francisco Javier Ruiz‐Sánchez:** Conceptualization; methodology; formal analysis; data curation; writing—review and editing. **Marta Elena Losa‐Iglesias**: Conceptualization; methodology; formal analysis; data curation; writing—review and editing. **Ricardo Becerro‐de‐Bengoa‐Vallejo**: Conceptualization; methodology; formal analysis; data curation; writing—review and editing. **Daniel López‐López:** Conceptualization; methodology; formal analysis; data curation; writing—review and editing. **Alonso Montiel‐Luque:** Conceptualization; methodology; formal analysis; data curation; writing—review and editing. **Carmen de Labra:** Conceptualization; methodology; formal analysis; data curation; writing—review and editing. **Miguel Ángel Saavedra‐García:** Conceptualization; methodology; formal analysis; data curation; writing—review and editing. **Emmanuel Navarro‐Flores:** Conceptualization; methodology; formal analysis; data curation; writing—review and editing.

## CONFLICT OF INTEREST STATEMENT

The authors declare that they have no conflicts of interest.

## ETHICS STATEMENT

The Bioethics and Biosafety Committee of the University of Valencia (Spain, 2020) approved this study (approval number 1450610). The participants signed the written consent before joining the research. The ethical and human experimentation standards of the Declaration of Helsinki (World Medical Association) and other organizations were always respected.

## Data Availability

The data that support the findings of this study are available from the corresponding author upon reasonable request.

## References

[1] Stack, E. L. , and H. C. Roberts 2013. “Slow Down and Concentrate: Time for a Paradigm Shift in Fall Prevention Among People With Parkinson’s Disease?” Parkinsons Disease 2013: 1–8. 10.1155/2013/704237. PMC3596919 23533952

[2] Lindholm, B. , P. Hagell , O. Hansson , and M. H. Nilsson . 2015. “Prediction of Falls and/or Near Falls in People With Mild Parkinson’s Disease.” PLoS One 10: e0117018. 10.1371/journal.pone.0117018. 25635687 PMC4311993

[3] Lieberman, A. , A. Deep , M. C. Olson , V. Smith Hussain , C. W. Frames , M. McCauley , and T. E. Lockhart . 2019. “Falls When Standing, Falls When Walking: Different Mechanisms, Different Outcomes in Parkinson Disease.” Cureus 11: 1–9. 10.7759/cureus.5329. PMC6777936 31598436

[4] LeWitt, P. A. , S. Kymes , and R. A. Hauser . 2020. “Parkinson Disease and Orthostatic Hypotension in the Elderly: Recognition and Management of Risk Factors for Falls.” Aging and Disease 11(3): 679–691. 10.14336/ad.2019.0805. 32489712 PMC7220277

[5] von Campenhausen, S. , B. Bornschein , R. Wick , K. Bötzel , C. Sampaio , W. Poewe , W. Oertel , U. Siebert , K. Berger , and R. Dodel . 2005. “Prevalence and Incidence of Parkinson’s Disease in Europe.” European Neuropsychopharmacology 15(4): 473–490. 10.1016/j.euroneuro.2005.04.007. 15963700

[6] García‐Ramos, R. , E. López Valdés , L. Ballesteros , S. Jesús , and P. Mir . 2016. “The Social Impact of Parkinson’s Disease in Spain: Report by the Spanish Foundation for the Brain.” Neurologia 31(6): 401–413. 10.1016/j.nrleng.2013.04.008. 23816428

[7] Lee, A. , and R. M. Gilbert . 2016. “Epidemiology of Parkinson Disease.” Neurologic Clinics 34(4): 955–965. 10.1016/j.ncl.2016.06.012. 27720003

[8] Ou, Z. , J. Pan , S. Tang , D. Duan , D. Yu , H. Nong , and Z. Wang . 2021. “Global Trends in the Incidence, Prevalence, and Years Lived With Disability of Parkinson’s Disease in 204 Countries/Territories From 1990 to 2019.” Frontiers in Public Health 9. 10.3389/fpubh.2021.776847. PMC8688697 34950630

[9] Pouwels, S. , M. T. Bazelier , A. De Boer , W. E. J. Weber , C. Neef , C. Cooper , and F. de Vries . 2013. “Risk of Fracture in Patients With Parkinson’s Disease.” Osteoporosis International 24(8): 2283–2290. 10.1007/s00198-013-2300-2. 23430103

[10] Huang, Y. F. , Y. G. Cherng , S. P. C. Hsu , C. C. Yeh , Y. C. Chou , C. H. Wu , T. L. Chen , and C. C. Liao . 2015. “Risk and Adverse Outcomes of Fractures in Patients With Parkinson’s Disease: Two Nationwide Studies.” Osteoporosis International 26(6): 1723–1732. 10.1007/s00198-015-3052-y. 25672807

[11] Kalilani, L. , M. Asgharnejad , T. Palokangas , and T. Durgin . 2016. “Comparing the Incidence of Falls/fractures in Parkinson’s Disease Patients in the US Population.” PLoS One 11(9): e0161689. 10.1371/journal.pone.0161689. 27583564 PMC5008740

[12] Allen, N. E. , A. K. Schwarzel , and C. G. Canning . 2013. “Recurrent Falls in Parkinson’s Disease: A Systematic Review.” Parkinson’s Disease 2013(1): 906274. 10.1155/2013/906274. PMC3606768 23533953

[13] Canning, C. G. , S. S. Paul , and A. Nieuwboer . 2014. “Prevention of Falls in Parkinson’s Disease: a Review of Fall Risk Factors and the Role of Physical Interventions.” Neurodegenerative Disease Management 4(3): 203–221. 10.2217/nmt.14.22. 25095816

[14] Kanegusuku, H. , R. M. Ritti‐Dias , P.Y. Igarasi Barbosa , E. T. das Neves Guelfi , E. Okamoto , C. S. Miranda , T. Paula Oliveira , and M. E. Pimentel Piemonte . 2021. “Influence of Motor Impairment on Exercise Capacity and Quality of Life in Patients With Parkinson Disease.” Journal of Exercise Rehabilitation 17(4): 241–246. 10.12965/jer.2142290.145. 34527635 PMC8413915

[15] Murueta‐Goyena, A , O. Muiño , and J. C. Gómez‐Esteban . 2024. “Prognostic Factors for Falls in Parkinson’s Disease: A Systematic Review.” Acta Neurologica Belgica 124(2): 395–406. 10.1007/s13760-023-02428-2. 38015306 PMC10965733

[16] Bowen, C. , A. Ashburn , M. Cole , M. Donovan‐Hall , M. Burnett , J. Robison , L. Mamode , R. Pickering , D. Bader , and D. Kunkel . 2016. “A Survey Exploring Self‐Reported Indoor and Outdoor Footwear Habits, Foot Problems and Fall Status in People With Stroke and Parkinson’s.” Journal of Foot and Ankle Research 9: 1–10. 10.1186/s13047-016-0170-5. 27688813 PMC5034630

[17] Antonini, A. , M. Tinazzi , G. Abbruzzese , A. Berardelli , K. R. Chaudhuri , G. Defazio , J. Ferreira , P. Martinez‐Martin , C. Trenkwalder , and O. Rascol . 2018. “Pain in Parkinson’s Disease: Facts and Uncertainties.” European Journal of Neurology 25(7): 917–924. 10.1111/ene.13624. 29520899

[18] Valkovic, P. , M. Minar , H. Singliarova , J. Harsany , M. Hanakova , J. Martinkova , and J. Benetin . 2015. “Pain in Parkinson´s Disease: A Cross‐Sectional Study of Its Prevalence, Types, and Relationship to Depression and Quality of Life.” PLoS One 10(8): e0136541. 10.1371/journal.pone.0136541. 26309254 PMC4550419

[19] Pouclet, H. , P. Derkinderen , and T. Lebouvier . 2013. “Foot Dystonia Heralding Levodopa‐Induced Dyskinesias in Parkinson Disease.” Clinical Neurology and Neurosurgery 115(2): 235–236. 10.1016/j.clineuro.2012.05.016. 22743235

[20] Jiménez‐Cebrián, A. M. , R. Becerro‐de‐Bengoa‐Vallejo , M. E. Losa‐Iglesias , C. de Labra , C. Calvo‐Lobo , P. Palomo‐López , E.M. Martínez‐Jiménez , and E. Navarro‐Flores . 2021. “Kinesiophobia Levels in Patients With Parkinson’s Disease: A Case‐Control Investigation.” International Journal of Enviromnmental Research and Public Health 18(9): 4791–4798. 10.3390/ijerph18094791. PMC8124702 33946205

[21] Yokote, A. , Y. Hayashi , S. Yanamoto , S. Fujioka , K. Higa , and Y. Tsuboi . 2022. “Leg Muscle Strength Correlates With Gait Performance in Advanced Parkinson Disease.” Internal Medicine 61(5): 633–638. 10.2169/internalmedicine.7646-21. 34393165 PMC8943390

[22] Aranda‐Gallardo, M. , A. Gonzalez‐Lozano , J. I. Oña‐Gil , J. M. Morales‐Asencio , A. Mora‐Banderas , and J. C. Canca‐Sanchez . 2022. “Relation between Hyponatraemia and Falls by Acute Hospitalised Patients: A Case‐Control Study.” Journal of Clinical Nursing 31(7–8): 958–966. 10.1111/jocn.15952. 34245058

[23] Kobylecki, C. , I. Shiderova , M. Boca , and E. Michou . 2022. “Falls Risk is Predictive of Dysphagia in Parkinson’s Disease.” Neurological Sciences 43(2): 1415–1417. 10.1007/s10072-021-05700-6. 34731336 PMC8789694

[24] Navarro‐Flores, E. , A. M. Jiménez‐Cebrián , R. Becerro‐de‐Bengoa‐Vallejo , C. Calvo‐Lobo , M. E. Losa‐Iglesias , C. Romero‐Morales , D. López‐López , and P. Palomo‐López . 2022. “Effect of Foot Health and Quality of Life in Patients With Parkinson Disease: A Prospective Case‐Control Investigation.” Journal of Tissue Viability 31(1): 69–72. 10.1016/j.jtv.2021.07.001. 34275724

[25] Jiménez‐Cebrián, A. M. , L. López‐López , M. E. Losa‐Iglesias , R. Becerro‐de‐Bengoa‐Vallejo , C. Romero‐Morales , D. López‐López , A. Montiel‐Luque , E. Navarro‐Flores , and C. de Labra . 2022. “The Implications of the Foot Health Status in Parkinson Patients: A Case–Control Study.” International Wound Journal 20: 100–108. 10.1111/iwj.13844. 35581151 PMC9797927

[26] Brognara, L. , E. Navarro‐Flores , L. Iachemet , N. Serra‐Catalá , and O. Cauli . 2020. “Beneficial Effect of Foot Plantar Stimulation in Gait Parameters in Individuals with Parkinson’s Disease.” Brain Sciences 10(2): 69. 10.3390/brainsci10020069. 32012779 PMC7071420

[27] Ketharanathan, T. , R. Hanwella , R. Weerasundera , and V. A. de Silva . 2014. “Major Depressive Disorder in Parkinson’s Disease: A Cross‐Sectional Study From Sri Lanka.” BMC Psychiatry 14: 278. 10.1186/s12888-014-0278-8. 25266218 PMC4188874

[28] Paker, N. , D. Bugdayci , G. Goksenoglu , D. T. Demircioğlu , N. Kesiktas , and N. Ince . 2015. “Gait Speed and Related Factors in Parkinson’s Disease.” Journal of Physical Therapy Science 27(12): 3675–3679. 10.1589/jpts.27.3675. 26834330 PMC4713769

[29] Garrow, J. S. 1986. “Quetelet Index as Indicator of Obesity.” Lancet (London, England) 1(8491): 1219. 10.1016/s0140-6736(86)91207-9. 2871462

[30] Hoehn, M. M. , and M. D. Yahr . 1967. “Parkinsonism: Onset, Progression, and Mortality.” Neurology 17(5): 427. 10.1212/wnl.17.5.427. 6067254

[31] Pagonabarraga, J. , J. Kulisevsky , G. Llebaria , C. García‐Sánchez , B. Pascual‐Sedano , and A. Gironell . 2008. “Parkinson’s Disease‐Cognitive Rating Scale: A New Cognitive Scale Specific for Parkinson’s Disease.” Movement Disorders 23(7): 998–1005. 10.1002/mds.22007. 18381647

[32] Fernández‐Bobadilla, R. , S. Martínez‐Horta , J. Marín‐Lahoz , A. Horta‐Barba , J. Pagonabarraga , and J. Kulisevsky . 2017. “Development and Validation of an Alternative Version of the Parkinson’s Disease‐Cognitive Rating Scale (PD‐CRS).” Parkinsonism & Related Disorders 43: 73–77. 10.1016/j.parkreldis.2017.07.015. 28754233

[33] Downton, J. 1993. Falls in the Ederly. London, UK: Arnold E.

[34] Aranda‐Gallardo, M. , J. M. Morales‐Asencio , J. C. Canca‐Sánchez , Á. Morales‐Fernández , A. B. Moya‐Suarez , A. M. Mora‐Banderas , C.,Pérez‐Jiménez , and Barrero‐Sojo, S. 2015. “Consequences of Errors in the Translation of Questionnaires: Spanish Version of Downton Index.” Revista de Calidad Asistencial 30(4): 195–202. 10.1016/j.cali.2015.04.003. 26068277

[35] Bueno‐García, M. J. , M. T. Roldán‐Chicano , J. Rodríguez‐Tello , M. D. Meroño‐Rivera , R. Dávila‐Martínez , and N. Berenguer‐García . 2017. “Características de la escala Downton en la Valoración del Riesgo de Caídas en Pacientes Hospitalizados.” Enfermia Clinica 27(4): 227–234. 10.1016/j.enfcli.2017.02.008. 28400165

[36] Vassallo, M. , L. Poynter , J. C. Sharma , J. Kwan , and S. C. Allen . 2008. “Fall Risk‐Assessment Tools Compared With Clinical Judgment: An Evaluation in a Rehabilitation Ward.” Age and Ageing 37(3): 277–281. 10.1093/ageing/afn062. 18456792

[37] López, M. , M. Fernández‐Castro , B. Martín‐Gil , M. F. Muñoz‐Moreno , and J. M. Jiménez . 2022. “Auditing Completion of Nursing Records as an Outcome Indicator for Identifying Patients at Risk of Developing Pressure Ulcers, Falling, and Social Vulnerability: An Observational Study.” Journal of Nursing Management 30(4): 1061–1068. 10.1111/jonm.13569. 35266605

[38] Wilczyński, J. , M. Ścipniak , K. Ścipniak , K. Margiel , I. Wilczyński , R. Zieliński , and P. Sobolewski . 2021. “Assessment of Risk Factors for Falls Among Patients with Parkinson’s Disease.” BioMed Research International 2021: 1–8. 10.1155/2021/5531331. 34621895 PMC8492255

[39] Sebastia‐Amat, S. , J. Tortosa‐Martínez , M. García‐Jaén , and B. Pueo . 2021. “Within‐Subject Variation in the Cognitive Timed up and Go Test as an Explanatory Variable in Fall Risk in Patients with Parkinson’s Disease.” Journal of Rehabilitation Medicine 53: jrm00234. 10.2340/16501977-2874. 34553234 PMC8638737

[40] Kegelmeyer, D. A. , A. D. Kloos , K. M. Thomas , and S. K. Kostyk . 2007. “Reliability and Validity of the Tinetti Mobility Test for Individuals With Parkinson Disease.” Physical Therapy 87(10): 1369–1378. 10.2522/ptj.20070007. 17684089

[41] Weil, R. S. , A. E. Schrag , J. D. Warren , S. J. Crutch , A. J. Lees , and H. R. Morris . 2016. “Visual Dysfunction in Parkinson’s Disease.” Brain 139(11): 2827–2843. 10.1093/brain/aww175. 27412389 PMC5091042

[42] Reina‐Bueno, M. , C. Calvo‐Lobo , D. López‐López , P. Palomo‐López , R. Becerro‐de‐Bengoa‐Vallejo , M. E. Losa‐Iglesias , C. Romero‐Morales , and E. Navarro‐Flores . 2021. “Effect of Foot Orthoses and Shoes in Parkinson’s Disease Patients: A Prisma Systematic Review.” Journal of Personalized Medicine 11: 1136–1145. 10.3390/jpm11111136. 34834488 PMC8621527

[43] Novo‐Trillo, E. , D. López‐López , C. de Labra , M. E. Losa‐Iglesias , R. Becerro‐de‐Bengoa‐Vallejo , C. Calvo‐Lobo , C. Romero‐Morales , and M. San‐Antolín‐Gil . 2020. “Impact of Footwear and Foot Deformities in Patients With Parkinson’s Disease: A Case‐Series Study.” International Journal of Medical Sciences 18: 372–377. 10.7150/ijms.50967 PMC7757138 33390806

[44] Mollà‐Casanova, S. , J. Pedrero‐Sánchez , M. Inglés , J. López‐Pascual , E. Muñoz‐Gómez , M. Aguilar‐Rodríguez , N. Sempere‐Rubio , and P. Serra‐Añó . 2022. “Impact of Parkinson’s Disease on Functional Mobility at Different Stages.” Frontiers in Aging Neuroscience 14: 935841. 35783141 10.3389/fnagi.2022.935841 PMC9249436

[45] Awale, A. , T. J. Hagedorn , A. B. Dufour , H. B. Menz , V. A. Casey , and M. T. Hannan . 2017. “Foot Function, Foot Pain, and Falls in Older Adults: The Framingham Foot Study.” Gerontology 63(4): 318–324. 10.1159/000475710. 28482340 PMC5501294

[46] Wylie, G. , C. Torrens , P. Campbell , H. Frost , A. L. Gordon , H. B. Menz , D. A. Skelton , F. Sullivan , M. D. Witham , and J. Morris . 2019. “Podiatry Interventions to Prevent Falls in Older People: A Systematic Review and Meta‐Analysis.” Age and Ageing 48(3): 327–336. 10.1093/AGEING/AFY189. 30615052 PMC6503946

